# New Trends in Vaccine Characterization, Formulations, and Development

**DOI:** 10.3390/vaccines12030338

**Published:** 2024-03-20

**Authors:** Ravinder Kumar

**Affiliations:** Department of Biological Sciences, Rensselaer Polytechnic Institute, Troy, NY 12180, USA; raj86tau@gmail.com

More than eight decades have passed since the development of the first vaccine in the 1940s. During all those decades, the world has seen the availability and clinical use of several dozen vaccines. Not only has the number of available vaccines increased, but the pace and volume of vaccine availability have increased significantly [1]. The scale on which vaccines were manufactured during the last few years was possible only due to newer vaccine development platforms and the increased number of production units spreading throughout the world [2]. Apart from conventional ways of vaccine development, which include the attenuation and inactivation of associated pathogens, modern-day vaccine formulation avoids the growth of pathogenic entities. Today’s vaccines, be they subunit, conjugate, VLPs, or mRNA-based, are much safer as these vaccines use only a few components of the pathogen, which rule out the possibility of vaccine-acquired infection and attenuated pathogens used as an immunogen in the vaccine transforming into a move infectious or virulent strain [3,4].

The use of newer platforms in vaccine formulation has also helped in the development of vaccines against diseases that were previously thought impossible. For example, a vaccine against malaria is possible only due to the use of a new platform for in-vaccine formulation. The protozoan’s surface protein (acting as an immunogen) was cloned, expressed, purified, and used as an immunogen to raise immunity [5]. It will be no surprise that the world may also see vaccine availability against leprosy and tuberculosis, again, all thanks to the modern-day practice of vaccine development [6]. Furthermore, the arrival of the new platform in vaccine development helps develop vaccines preventing cancer. For example, a VLPs-based vaccine formulation under the trade name Gardisal9 is in clinical use against human papilloma. Apart from this, several other VLPs-based vaccines have been approved for clinical use [7].

The most recent and newest addition in vaccine formulation is the use of nucleic acid-based vaccines during the COVID-19 pandemic. The mRNA-based vaccine against coronavirus introduced by Pfizer and Moderna towards the end of 2020 can be seen as one of the most significant breakthroughs in vaccine development [8]. The worldwide use of mRNA-based vaccines during the pandemic showed the technology’s safe and reliable nature. This is important as it shows the public’s acceptance of technology and boosts more research and development in mRNA-based vaccines [9]. Surprisingly, this technology has gained significant importance in vaccine development. As a result, in a short span of a few years, several mRNA-based vaccines have entered different phases of clinical trials [10].

Another important and likely platform for vaccine development and formulation is the use of whole recombinant yeast as a micro container for the storage and delivery of immunogen/drugs [11]. This platform offers several advantages, including the long-term stability of immunogen at ambient temperatures and the stability of immunogen during freeze and thaw processes. This whole recombinant yeast approach showed promising results in both pre-clinical and clinical trials [12]. A further continuous rise in anti-fungal resistance and global fungal burden pushed the requirement for an anti-fungal vaccine. Even in this case, using inactivated whole yeast or recombinant yeast can significantly help. Several pre-clinical studies have shown the ability of inactivated yeast to raise a protective immune response against fungal infection [13,14].

The deployment of new platforms in vaccine development and formulation allows for the development of safe, effective, and large volumes of vaccines in a short duration. Despite this, vaccines developed using different regimes suffer from common problems, which include poor or short shelf life at ambient temperatures and the need for a continuous cold chain during transportation, storage, and final distribution before final administration [15]. Therefore, the focus of vaccine formulation should also be on improving the stability of vaccines and ways to prevent the need for a cold chain. Several methods have been tested; some look promising [16,17,18,19,20]. Owing to new and modern challenges in the form of geopolitics (wars, sanctions), vaccine manufacturers and developers should also pay attention to these issues [21]. Whether a given approach is specific to a given vaccine or formulation or suitable for different vaccine formulations must be tested [22].

Therefore, the world has come a long way in terms of vaccine characterization, formulations, and development; however, there are still many challenges that need to be taken care of if the world wants to make the best use of available vaccines in all socioeconomic settings, communities, or societies. Further, all efforts should be made to ensure that vaccines are available to each individual on the planet by keeping socioeconomic and geopolitics issues separate. Issues like vaccine hoarding or vaccine monopoly should be dealt with in a more appropriate way by keeping it more human-centric [23]. Therefore, apart from an improvement in the manufacturing process, we need to work on other issues (mentioned above) to make the best use of all the available vaccines.
